# Clinical Use of Apis mellifera Honey Membrane in Second- and Third-Degree Burns: A Retrospective Case Series

**DOI:** 10.7759/cureus.104824

**Published:** 2026-03-07

**Authors:** Jeane Fuenmayor, Víctor M Rivero-Díaz

**Affiliations:** 1 Plastic and Reconstructive Surgery, Instituto Guatemalteco de Seguridad Social, Mixco, GTM; 2 Faculty of Medical Sciences, Universidad de San Carlos de Guatemala, Guatemala City, GTM

**Keywords:** apis mellifera, biological dressing, case report series, full-thickness burn, honey, honey dressing, partial-thickness burn, skin graft, ­wound healing

## Abstract

Background: Burn management requires cost-effective, antimicrobial dressings, especially in resource-limited settings. We evaluated the clinical performance of a unilaminar *Apis mellifera* honey membrane in treating adult second- and third-degree burns.

Methods: A retrospective case series was conducted at a tertiary hospital. Ten adults with second-degree (n = 5, 50%) and third-degree (n = 5, 50%) burns received unilaminar honey membrane (Bio-film®, Biomed Centroamérica, Guatemala City, Guatemala) application over the affected area.

Results: Ten patients (nine males and one female; mean age of 44.2 years) were included. Etiologies comprised scalds (5, 50%), fire (2, 20%), electrical (2, 20%), and contact (1, 10%) burns. A total of 11 lesions were treated. Second-degree burns achieved complete closure with a single application in a mean of 7.4 days (95% CI: 5.3-9.5). Third-degree burns required two to four applications to achieve a graft-ready bed in a mean of 17.7 days (95% CI: 13.4-22.0). All skin grafts (100%) integrated successfully. Tolerance was excellent, with only one patient reporting transient pain. Pre-graft cultures showed superficial colonization in two (33%) of the six deep wounds, with no signs of clinical infection.

Conclusions: The unilaminar *Apis mellifera* honey membrane appeared to be a safe and well-tolerated adjunct for second-degree burn closure and graft-bed preparation. While preliminary results are promising, further large-scale comparative studies are required to confirm its clinical efficacy and cost-effectiveness.

## Introduction

Burn injuries represent a major public health concern due to their high morbidity, infection risk, and the functional and aesthetic sequelae they cause. Appropriate local management is essential to promote rapid healing, reduce pain, and prevent infectious complications. In this context, the use of natural products with antimicrobial and biostimulatory properties has generated growing clinical and scientific interest.

Honey from *Apis mellifera* has been used for therapeutic purposes since ancient times and, in recent decades, has become the focus of numerous studies for its ability to promote wound healing and control bacterial bioburden. Meta-analyses and systematic reviews confirm that honey-based dressings significantly accelerate healing time and reduce pain compared to conventional treatments such as silver sulfadiazine, achieving complete epithelialization in shorter periods and with fewer dressing changes [1-3]. This clinical efficacy is attributed to complementary mechanisms of action: hydrogen peroxide production by the enzyme glucose oxidase, acidic pH, and osmotic pressure that inhibit bacterial growth, as well as the presence of phenolic compounds, flavonoids, and antimicrobial peptides (defensin-1) that enhance antimicrobial and antioxidant activity [4-9]. Moreover, honey modulates the inflammatory response, promotes angiogenesis, and stimulates keratinocyte and fibroblast migration, facilitating granulation tissue formation and re-epithelialization [6].

In patients with partial- and full-thickness burns, these properties suggest that honey may serve as an alternative or adjunct to conventional dressings, particularly in a context of increasing antimicrobial resistance and the need for cost-effective therapies.

The objective of this study is to evaluate the clinical efficacy of a unilaminar *Apis mellifera *honey membrane in the treatment of second- and third-degree burns in adults, assessing time to epithelialization or graft-bed readiness, dressing change frequency, post-application pain, and infection occurrence.

## Materials and methods

Study design and participants

This study was a retrospective case series conducted at the burn unit of a tertiary referral hospital between September 2021 and February 2022. We reviewed the medical records of all patients who received the intervention during the study period. Eligible participants were adult patients (≥18 years) with a clinical diagnosis of second- or third-degree burns requiring hospital management, including thermal, electrical, or contact burns, regardless of anatomical location. Patients were excluded if they had a known allergy to bee-derived products, were pregnant, or suffered from uncontrolled severe immunosuppression.

Intervention and clinical protocol

The intervention consisted of a unilaminar membrane based on *Apis mellifera* honey (Bio-film®, Biomed Centroamérica, Guatemala City, Guatemala). The product is a biological/epithelial bioregenerative dressing composed primarily of purified *Apis mellifera* honey (approximate pH of 3.1) combined with acetic acid, policresulen, and fenugreek (*Trigonella foenum-graecum*) seed extract [10].

Standard wound preparation involved cleansing with sterile saline solution and gentle mechanical debridement when necessary. The membrane was placed directly over the wound bed, covered with a non-adherent mesh and sterile gauze, and secured with an elastic bandage. Treatment protocols were stratified by burn depth. For second-degree burns, a single application was maintained until complete closure. Conversely, third-degree burns underwent application following escharotomy and/or fasciotomy, with membrane replacements every six days until a red, flat, infection-free bed suitable for grafting was achieved. All patients received care according to burn severity and in compliance with the institutional guidelines of the Guatemalan Social Security Institute [11]. No experimental interventions were performed beyond standard care.

Outcome variables and data analysis

Key outcomes included time to epithelialization or graft-bed readiness (days), the number and frequency of membrane replacements, and post-application pain measured with a modified visual analog scale (0-10). Presence of infection was determined by tissue culture prior to the first application and before grafting in third-degree burns, where colonization was defined as bacterial growth without clinical signs of local or systemic infection.

A descriptive analysis was performed. Categorical variables were expressed as absolute frequencies and percentages (n, %). Continuous variables were reported as mean and range. Given the sample size (n = 10), 95% confidence intervals (CI) were calculated for continuous outcome variables using the t-distribution method. The unit of analysis for clinical outcomes was the specific burn wound, distinguishing between second- and third-degree lesions in patients with mixed injuries. Data were recorded and analyzed using Microsoft Excel (Microsoft Corporation, Redmond, WA). No data imputation was performed as there were no missing values.

Ethics

This study was approved by the IRB (approval number: 002406) and was conducted in accordance with the Declaration of Helsinki.

## Results

Patient characteristics and demographics

A total of 10 adult patients were included in the analysis, comprising nine (90%) males and one (10%) female, with a mean age of 44.2 years (95% CI: 28.1-60.3). Regarding etiology, scalds from hot liquids were the most frequent cause, affecting five (50%) patients, followed by high-voltage electrical burns in two (20%), flame burns in two (20%), and contact burns in one (10%). Comorbidities were present in three (30%) patients, including arterial hypertension and type 2 diabetes mellitus. Clinical characteristics and outcomes are summarized in Table 1.

**Table 1:** Patient characteristics and clinical outcomes. * Superficial colonization. TBSA: total body surface area; KDIGO: Kidney Disease Improving Global Outcomes.

No.	Age	Sex	Etiology	Burn degree	Anatomical location	TBSA %	No. of Biofilm® applications	Days to closure or graft bed	Pre-graft culture	Pain modified visual analog scale (0-10)	Comorbidities
1	38	M	Scald	II	Thorax and right upper limb	6.5%	1	7	–	0	Uncontrolled arterial hypertension
2	29	M	Hot oil	II	Right forearm	1.8%	1	6	–	0	–
3	82	M	Hot coffee	II	Right hand	0.5%	1	6	–	6	–
4	31	M	Hot coffee	II	Anterior thorax	6%	1	8	–	0	–
5	22	M	Fire	II–III	Bilateral upper limbs	18%	1 (II)/2 (III)	10 (II)/12 (III)	Negative	0	–
6	69	M	Electrical	III	Hands and forearms (bilateral)	8%	3	18	Positive*	0	Arterial hypertension, type 2 diabetes mellitus
7	51	F	Hot liquids	III	Dorsum of the left foot	1.5%	4	24	Negative	0	Type 2 diabetes mellitus, renal insufficiency, femoral and tibial artery obstruction (70%) of the left lower limb, cellulitis
8	71	M	Contact with hot sand	III	Bilateral plantar surfaces	4%	3	16	Negative	0	Diabetes mellitus, renal insufficiency KDIGO 2
9	22	M	Fire	III	Right wrist	0.5%	3	16	Positive*	0	–
10	27	M	Electrical	III	Left upper limb	9%	3	20	Negative	0	–

Clinical outcomes in second-degree burns

**Figure 1:**
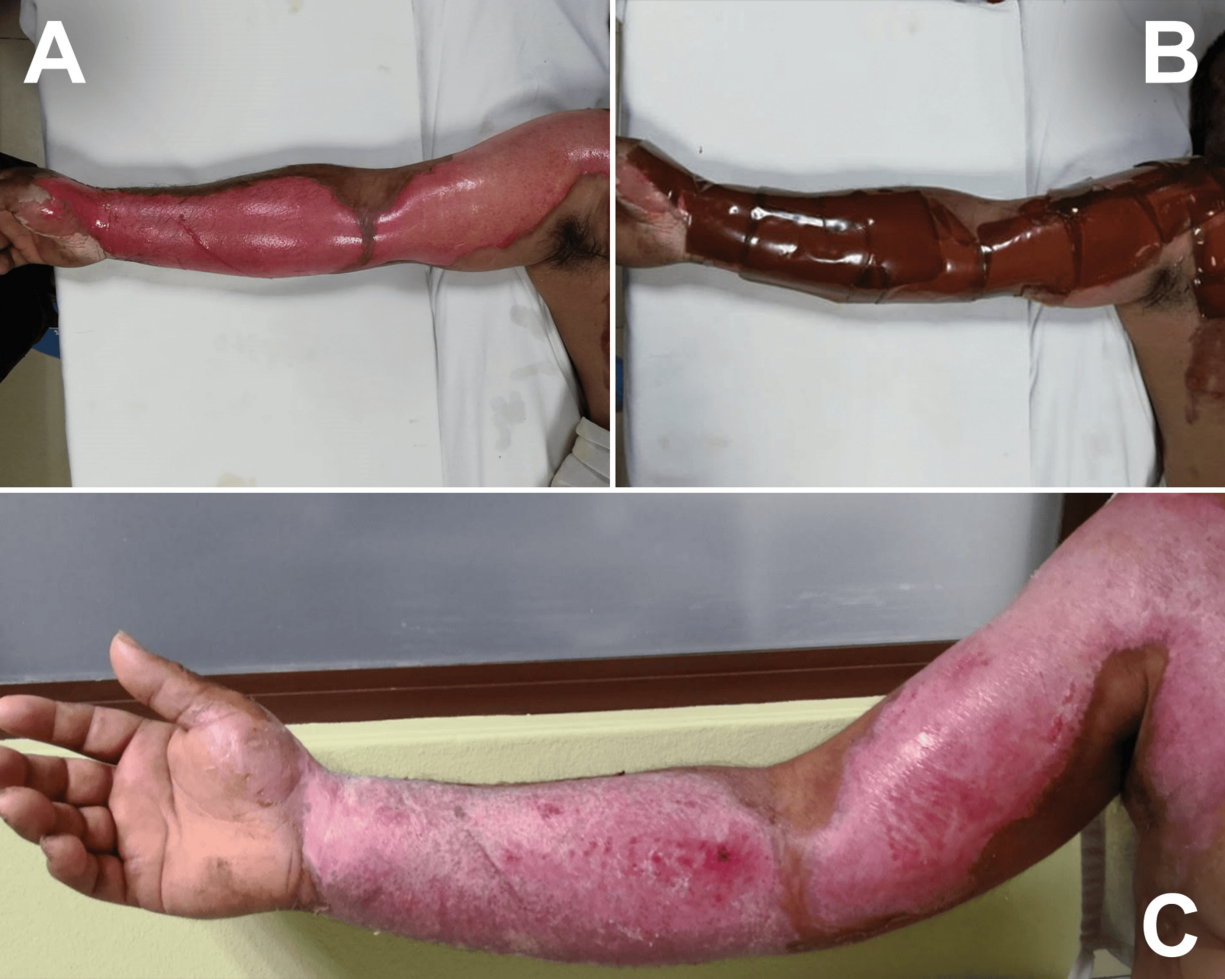
Superficial and deep second-degree scald burn. Superficial and deep second-degree scald burn with hot water on the right upper limb (A). Topical application of unilaminar *Apis mellifera* honey membrane (Bio-film®) over the affected area, with a superficial mesh placed on top to allow molding and adaptation of the biofilm to the wound surface (B). Completely epithelialized area after treatment, showing pink coloration and residual hypopigmentation, consistent with recent epithelialization and the early phase of skin remodeling (C).

Five lesions were classified as second-degree burns. In these cases, the treatment protocol consisted of a single Bio-film® application, which remained in place for six to 10 days. Complete wound closure was achieved in 100% of these cases, with a mean healing time of 7.4 days (95% CI: 5.3-9.5). As illustrated in Figure 1, the membrane demonstrated high conformability to the wound bed, facilitating epithelialization. Post-treatment assessment showed pink coloration consistent with recent epithelialization and early remodeling.

Management of third-degree burns

**Figure 2:**
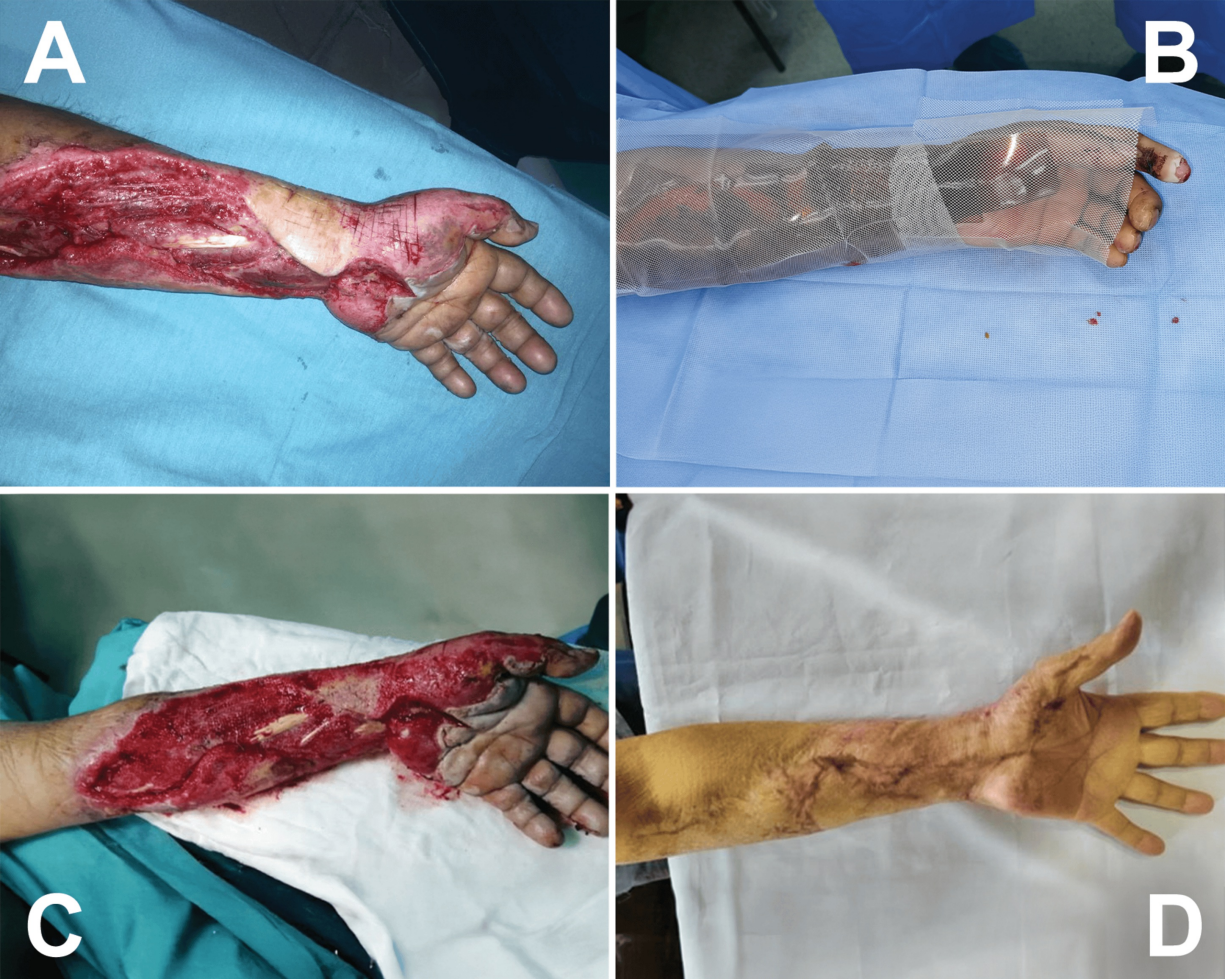
Third-degree high-voltage electrical burn. Immediate appearance following emergency fasciotomy for high-voltage electrical burn (A). Topical application of unilaminar *Apis mellifera* honey membrane (Bio-film®) over the surgical bed (B). Clean, flat, bright red wound bed without active bleeding (C). Functional and aesthetic outcome at six months, with restoration of mobility in the affected hand (D).

Six lesions were classified as third-degree burns, requiring surgical intervention prior to membrane application. These patients underwent escharotomy or fasciotomy as indicated, followed by Bio-film® applications every six days. Patients required between two and four sessions to achieve a viable wound bed. The mean time to graft-bed readiness was 17.7 days (95% CI: 13.4-22.0). High-voltage electrical injuries generally require a higher number of applications compared to thermal burns. Figure 2 demonstrates the progression of a high-voltage electrical burn from immediate post-fasciotomy status to a clean, granulated bed. Following preparation, 100% of patients underwent split-thickness skin grafting with successful integration.

Safety and tolerability

The safety profile was favorable across the cohort. Post-application pain was minimal; only one (10%) patient reported moderate pain (6/10 on the visual analog scale), which resolved within 24 hours with standard oral analgesia. Microbiological surveillance via tissue cultures revealed negative results in four (67%) of the six third-degree burn patients. In the remaining two (33%) cases, one electrical and one flame burn, superficial colonization was detected; however, no patients developed clinical signs of local or systemic infection.

## Discussion

In this case series comprising 10 adult patients with a total of 11 treated lesions (five (45%) second-degree and six (55%) third-degree), the use of the unilaminar *Apis mellifera* honey membrane (Bio-film®) was associated with favorable outcomes. Specifically, the intervention facilitated complete closure of second-degree burns with a single application within six to 10 days and effectively prepared graft beds in third-degree burns over two or three sessions. Graft integration was successful in 100% of cases, and post-application pain was reported by only one patient (1, 10%), resolving rapidly. These findings confirm the potential of honey as an effective, well-tolerated, and easy-to-use therapeutic option for burn management.

The results are consistent with meta-analyses and systematic reviews reporting that honey-based dressings accelerate wound healing, reduce pain, and achieve early wound sterilization compared to silver sulfadiazine and other conventional treatments [1-3]. In controlled studies, honey has demonstrated increased epithelialization rates, reduced bacterial load, and shorter hospital stays, with an excellent safety profile [2,4]. Our experience adds evidence supporting its adjuvant use in full-thickness burns, where rapid achievement of a well-vascularized, infection-free bed is critical for successful grafting.

The biological framework underlying these results is extensive. Honey exerts antimicrobial effects through hydrogen peroxide generation, acidic pH, high osmotic pressure, and the presence of phenolic compounds and antimicrobial peptides such as defensin-1, which damage bacterial membranes and promote re-epithelialization [4-7]. Additionally, it modulates the inflammatory response by reducing pro-inflammatory cytokines (tumor necrosis factor-alpha (TNF-α), IL-1β, IL-6) and promoting the transition from M1 to M2 macrophages, thereby stimulating the proliferative phase of wound healing. It also induces the production of growth factors (vascular endothelial growth factor (VEGF), fibroblast growth factor (FGF), epidermal growth factor (EGF), transforming growth factor-beta (TGF-β)) and the synthesis of type I and III collagen, facilitating angiogenesis and granulation tissue formation [6,8,9].

From a clinical standpoint, the reduced number of dressing changes and improved pain control observed in our series may translate into decreased healthcare burden, better patient experience, and potential cost reduction, findings also reported by other authors [12,13]. These advantages, combined with activity against multidrug-resistant bacterial strains and prevention of biofilm formation [5], make the honey membrane an especially appealing alternative in the context of rising antimicrobial resistance.

Strengths and limitations

The main strengths of this study include the standardized application of the treatment protocol with defined intervals and consistent microbiological evaluation in a consecutive cohort of real-world patients. Limitations include the small sample size, non-comparative design, and single-center setting, which restrict the generalizability of the findings. Outcomes were assessed by the treating team, which may introduce observer bias. The retrospective nature of the study may involve information bias due to reliance on existing medical records. Comparative and controlled trials are needed to confirm these results and further explore outcomes such as scar quality and cost-effectiveness.

## Conclusions

In this retrospective case series, the unilaminar *Apis mellifera* honey membrane appeared to be a safe and well-tolerated adjunct for burn management. Our findings indicate that this biological dressing facilitates rapid epithelialization in second-degree burns with a single application and effectively prepares the wound bed for successful grafting in third-degree injuries. Notably, the intervention was associated with high graft integration rates, minimal patient-reported pain, and a manageable safety profile with no evidence of invasive infection. These clinical advantages, combined with the reduced frequency of dressing changes, suggest that the honey membrane offers a practical alternative to conventional treatments, potentially lowering the burden of care. While larger, controlled trials are necessary to strictly validate efficacy and cost-effectiveness, the current results support its integration into burn management protocols, particularly in resource-limited settings where optimizing therapeutic efficiency is critical.
